# 

**Published:** 1890-08

**Authors:** 


### CONTENTS.

ORIGINAL COMMUNICATIONS—

Twelve Consecutive Laparotomies. By Virgil O. Hardon, M. D., Atlanta, Ga. .... 321
The Importance of Early Treatment in Middle Ear Disease. By N. C. Steele, M. D., Chattanooga, Tenn. .... 329
A Case of Generalized Scleroderma. By M. B. Hutchins, M. D., Atlanta, Ga. .... 333
Therapeutical Notes on Cascara Sagrada. By C. E. Murphey, M. D., Atlanta, Ga. .... 335
Report of Exsection of Shoulder. By C. H. Cox, M. D., Savannah, Ga. .... 337
Medical Corps of the Confederate Army and Navy, 1861–1865. Official Report by Joseph Jones, M D., of New Orleans, La. .... 339
The Scraping Out (Curetting) of Chancroid. By. O. Peterson, M. D., St. Petersburg .... 353

CORRESPONDENCE—

Our New York Letter .... 359

SOCIETY REPORTS—

Allegheny County Medical Society .... 364

EDITORIAL—

Doctors Who Advertise .... 372
Dr. R. C. Word .... 374
Resolutions of Respect to the Memory of Dr. Willis F. Westmoreland .... 375
Tribute to the Memory of Dr. W. D. Bizzell .... 376

SELECTIONS—

Uterine Treatment, the Abuse of .... 377
Collegiate Education and the Medical Profession .... 380
A New Method of Artificial Respiration .... 381
Turpentine in Post-Partum Hemorrhage .... 382
Sterilization of Water .... 383
Cerebral Surgery .... 383
Index to Advertisements on Page xl.
Reading Notices on Page xxxiv.
Our Premium List on Page xliv.

